# Effect of Freezing Process on the Microstructure of Gelatin Methacryloyl Hydrogels

**DOI:** 10.3389/fbioe.2021.810155

**Published:** 2021-12-14

**Authors:** Taotao Liu, Yuzhuo Zhang, Mingyue Sun, Meiqi Jin, Wei Xia, Huazhe Yang, Tianlin Wang

**Affiliations:** ^1^ School of Intelligent Medicine, China Medical University, Shenyang, China; ^2^ Changchun Medical College, Changchun, China

**Keywords:** GelMA, freezing temperature, freezing time, morphology, cell proliferation

## Abstract

Gelatin methacryloyl (GelMA) hydrogels have aroused considerable interests in the field of tissue engineering due to tunable physical properties and cell response parameters. A number of works have studied the impact of GelMA concentration, photo-initiator concentration, methacrylic anhydride (MA) concentration, cooling rate and temperature gradient on GelMA hydrogel generation, but little attention has been paid to the effect of the freezing temperatures and freezing time of GelMA prepolymer solution during preparation. In this study, GelMA hydrogels were synthesized with different freezing temperatures and time. It was found that the lower freezing temperatures and longer freezing time caused smaller pore sizes that realized higher cell viability and proliferation of MC3T3-E1 cells. The results showed that tunable microstructure of GelMA could be achieved by regulating the freezing conditions of GelMA, which provided a broad prospect for the applications of GelMA hydrogels in tissue engineering.

## Introduction

In recent years, many studies have focused on gelatin methacryloyl (GelMA) hydrogels, owing to the tunable physicochemical properties and good biocompatibility of the hydrogel (Yue et al., 2015; Sun et al., 2018; Basara et al., 2019; Liu et al., 2020; Elkhoury et al., 2021). GelMA hydrogel is a kind of photopolymer which is produced by the introduction of photosensitive groups on the side chains of gelatin (Lee et al., 2015a; Li et al., 2016; Sun et al., 2018). Solid GelMA hydrogel with good thermal stability can be obtained by photocrosslinking of GelMA prepolymer solution (Zhao et al., 2016; Kirsch et al., 2020). GelMA hydrogels possess arginine-glycine-aspartic acid (RGD) peptide sequences and matrix metalloproteinase (MMP) sequences, which favor cell adhesion and remodeling (Yue et al., 2015; Sun et al., 2018; Basara et al., 2019). Moreover, the porosities of GelMA hydrogels play an important role in the transport of oxygen and nutrients required by cells (Van Vlierberghe et al., 2007). Also, it has been demonstrated that the GelMA hydrogels at a relatively low concentration (i.e., ≤5% w/v) was more conducive for cell growth (Yin et al., 2018; Basara et al., 2019), but low concentration would lead to low compression modulus (Celikkin et al., 2018). Therefore, it is important to find a balance between supporting cell growth and possessing adequate mechanical properties since GelMA hydrogels have been widely used in tissue engineering (Zhang et al., 2018; Zheng et al., 2018; Wang et al., 2019). However, it is still a challenge to produce GelMA hydrogel scaffolds with proper pore sizes and mechanical properties for various tissue engineering applications (Celikkin et al., 2018; Zhang et al., 2018; Zheng et al., 2018; Gao et al., 2019; Wang et al., 2019).

One way to control the pore sizes of GelMA hydrogels is to adjust the concentration of GelMA or photo-initiator. For instance, Lee *et al.* (Lee et al., 2015b) and Celikkin *et al.* (Celikkin et al., 2018) confirmed that pore sizes would be affected by polymer concentration. Benton *et al.* (Benton et al., 2009) tuned the porosities, pore sizes and wall thicknesses of GelMA hydrogels indirectly by varying the photo-initiator concentration. Another way to change the pore sizes is to introduce other materials. For example, Wang *et al.* (Wang et al., 2014) combined GelMA with dextran glycidyl methacrylate (DexMA) to synthesize GelMA–DexMA copolymer hydrogels. They found that the pore sizes of GelMA–DexMA copolymer hydrogels would reduce as the degree of substitution (DS) of DexMA increased. In addition, the concentration of methacrylic anhydride (MA), the cooling rate and the temperature gradient could also affect pore sizes of GelMA hydrogels (Van Vlierberghe et al., 2007). Although the effects of these factors on the pore sizes of GelMA hydrogels have been demonstrated, the effect of the freezing temperatures and freezing time of GelMA prepolymer solution, which may have significant impacts on the pore sizes of the hydrogels, seem to be ignored.

In this study, the freezing temperatures and freezing time of GelMA prepolymer solution were studied and the influences of freezing conditions on pore sizes were evaluated. The effect of distinct pore sizes on swelling properties and mechanical properties was also investigated. Moreover, MC3T3-E1 cells were encapsulated in the GelMA hydrogels to study the cell proliferation.

## Materials and Methods

### Materials

Gelatin (biochemical reagent) and 2-hydroxy-4′-(2-hydroxyethoxy)-2-methylpropiophenone (Irgacure 2,959, 98%) were purchased from Shanghai Yuanye Biotechnology Co., Ltd. Phosphate buffered saline (PBS, pH = 7.4, without calcium and magnesium) was purchased from Biological Industries. Methacrylic anhydride (MA, 94%) was purchased from Sigma-Aldrich. DME/F-12 1:1 (1X), fetal bovine serum (FBS), penicillin- streptomycin solution and trypsin 0.25% (1X) solution were purchased from Hyclone. 4% tissue cell fixative was purchased from Solarbio.

### Synthesis of GelMA Prepolymers

The synthesis of gelatin methacryloyl (GelMA) hydrogel has been reported previously (Van den Bulcke et al., 2000; Chen et al., 2012). Briefly, GelMA prepolymers with different freezing temperatures and time were synthesized as follow: 20 g of gelatin was added into 200 ml of PBS and dissolved by stirring with a magnetic stirrer at 50°C and 240 rpm until completely dissolved. Then, 16 ml of methacrylic anhydride (MA) was added dropwise at 0.2 ml/min by using a micro syringe pump to the dissolved gelatin solution under continuous stirring. The mixed solution was allowed to react for 2 h by stirring at 50°C and 240 rpm using a magnetic stirrer. 50°ml of PBS was preheated to 50°C and then added into the mixed solution. After magnetic stirring for 10 min at 50°C, the reaction solution was placed in dialysis tubing and dialyzed in deionized water by stirring at 40°C and 500 rpm for 10 days to remove salts and unreacted MA. After dialysis, 400 ml of deionized water was added into the dialytic solution, heated to 40°C and stirred for 15 min. Finally, the solution was packed in 5 ml centrifuge tubes, and placed at −20°C, −40°C and −80°C for 2, 7, 12 and 17 days, respectively. Then, these samples were lyophilized for 4 days to obtain GelMA prepolymer (white foam) and stored in a drying dish at room temperature before use. In the end, 12 groups of samples were obtained with detailed freezing information listed in Table 1.

**TABLE 1:** The 12 groups of samples with different freezing temperatures and time.

Group	Temperature (°C)	Time (day)
1#	−20	2
2#	−20	7
3#	−20	12
4#	−20	17
5#	−40	2
6#	−40	7
7#	−40	12
8#	−40	17
9#	−80	2
10#	−80	7
11#	−80	12
12#	−80	17

### Preparation of GelMA Hydrogels

Freeze-dried GelMA prepolymers with different freezing temperatures and time were dissolved in PBS containing 1% (w/v) 2-hydroxy-4′-(2-hydroxyethoxy)-2-methylpropiophenone (Irgacure 2,959, 98%) as photoinitiator at room temperature and mixed by ultrasound. Then, the prepared solution was transferred to 2 ml centrifuge tube caps and exposed to ultraviolet (UV) light of 365 nm for 5 min. Finally, group 1#, 2#, 3# and 4# were frozen at −20°C, group 5#, 6#, 7# and 8# were frozen at −40°C, group 9#, 10#, 11# and 12# were frozen at −80°C for 6 h, respectively, and then lyophilized overnight to generate lyophilized GelMA hydrogels.

### Fourier-Transform Infrared Spectroscopy

Fourier-transform infrared (FTIR) spectra of Freeze-dried GelMA hydrogels were recorded with a FTIR spectrometer (FTIR, Agilent Cary 630, United States). The samples and the KBr powder were mixed, and poured into a tableting mold to be pressed into a sheet. Every measurement includes 128 scans, 4cm^−1^ resolution and the Y-axis format for transmittance (Rahali et al., 2017).

### Scanning Electron Microscope

The surface morphology of lyophilized GelMA hydrogels that had been sprayed with platinum was obtained from a scanning electron microscope (SEM, JSM-IT200, Japan) at 10 kV. The average pore size was analyzed with the SEM images (DursunUsal et al., 2019). No less than 30 pores were selected manually and randomly for each sample (Wang et al., 2018).

### Swelling Ratio

Freeze-dried GelMA hydrogels were weighted to obtain the dry weight of GelMA hydrogels, as dryweight (*W*_d_). Then, lyophilized GelMA hydrogels were immersed in PBS at room temperature, and the weight of GelMA hydrogels was measured after swelling for 30 min, 1, 2, 4, 8, 12, 24, and 48 h (the excess PBS on the sample surface was dried with filter paper before weighing), as wetweight (*W*_s_).

The swelling ratio was calculated by the following equation (Rahali et al., 2017; Liu et al., 2019):

$$\text{swelling}\;\text{ratio}=\frac{W_{\text{s}}-W_{\text{d}}}{W_{\text{d}}}\times 100\text{\%}$$ (1)

### Mechanical Properties

The mechanical properties of samples reaching swelling balance were tested using a biomechanical testing machine (Qixiang QX-W100, China). The samples were tested at a speed rate of 1 mm/min at room temperature. The compressive modulus was calculated from the slope of the linear region of the stress-strain curve that corresponds to 0–10% strain.

### Cell Culture

2 × 10^5^ MC3T3-E1 cells were encapsulated in different groups of GelMA (7.5 %) hydrogel-based scaffolds. After 1, and 3 days, the viability of cells was assessed by using AO/PI assay (Acridine Orange and Propidium Iodide, BestBio BB-4126-100 T). At each day after removing media, scaffolds were washed with PBS twice, and the AO and PI (Acridine Orange and Propidium Iodide) were mixed and added to each well and incubated for 20 min. Cell proliferation in different samples were detected using a laser scanning confocal microscope (LSCM, Zeisee LSM880).

### Data Analysis

All results in this study were expressed as mean ± standard deviation. Data analysis was carried out by Student’s *T* test and one-way analysis of variance (ANOVA), and the level of significance was set at *p* < 0.05.

## Results and Discussion

### Component of GelMA Hydrogels

**FIGURE 1:**
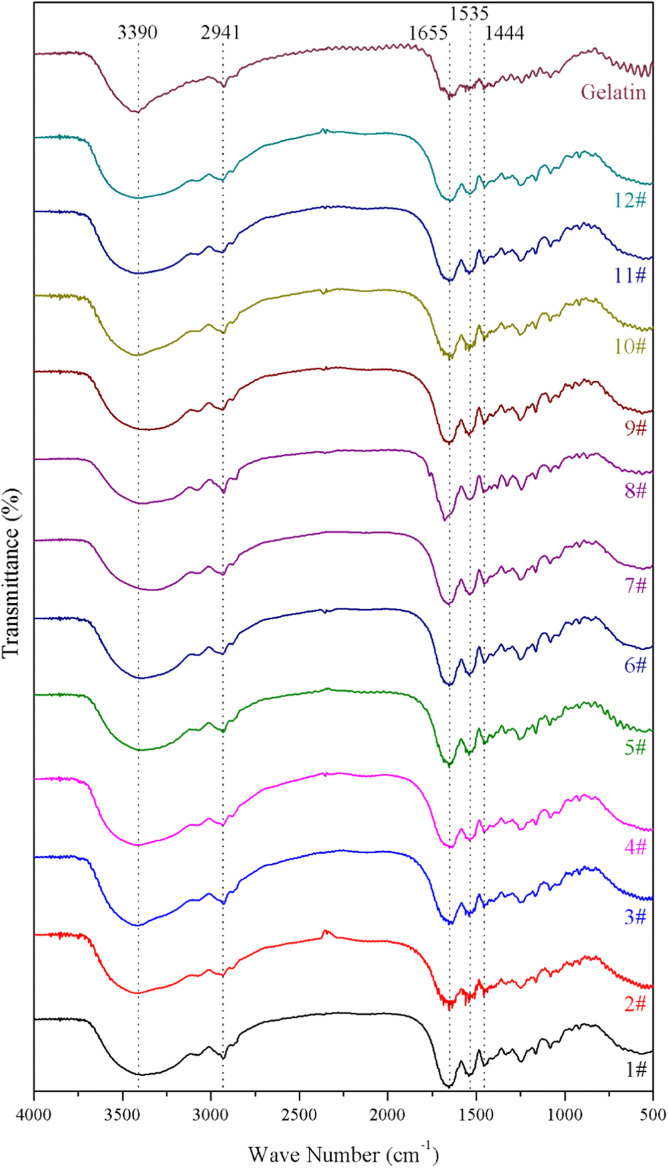
The fourier-transform infrared (FTIR) spectra of the GelMA hydrogels with different freezing temperatures and time.

**FIGURE 2:**
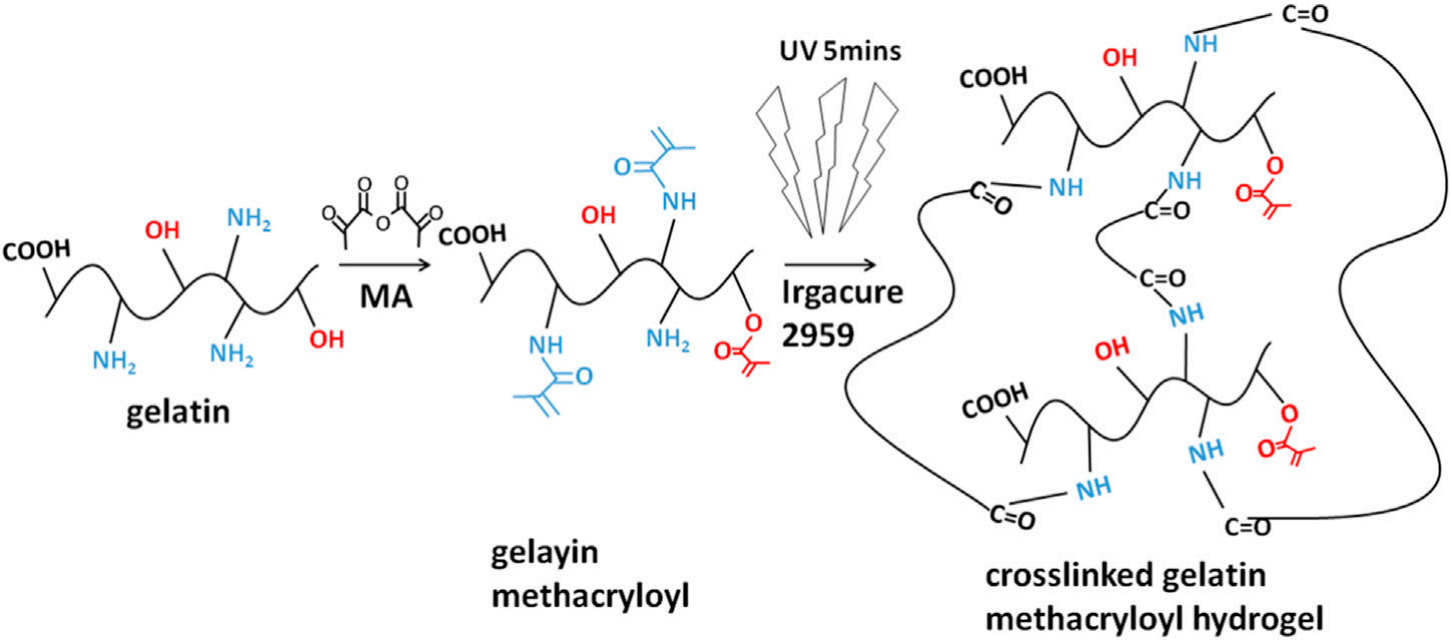
The molecular formula of protein/peptide (gelatin) and MA-modified protein/peptide (GelMA).

The fourier-transform infrared (FTIR) spectra of the GelMA hydrogels with different freezing temperatures and time are shown in Figure 1. According to the molecular formula (Figure 2) of gelatin and GelMA of the previous paper (Elkhoury et al., 2021) as can be seen from FTIR spectra, the absorption patterns of all samples were similar. The stretching vibration peak of -OH groups was observed at 3,305 cm^−1^. Owing to the presence of methacryloyl groups in GelMA hydrogels, characteristic peaks of amide were observed. A strong absorption peak emerged at 1,655 cm^−1^, which was due to the C=O stretching vibration of amide functional groups. The peak at 3,305 cm^−1^ was also contributed by the N-H stretching vibration, and the peak at 1,535 cm^−1^ was attributed to the N-H bending vibration. The peak at 2,941 cm^−1^ was due to the stretching vibration of C-H and the peak at 1,444 cm^−1^ represented the bending vibration of C-H (Sadeghi and Heidari, 2011; Rahali et al., 2017). Therefore, gelatin had been modified by MA successfully (Sadeghi and Heidari, 2011). According to the FTIR spectra, the chemical structure of GelMA hydrogels was not changed under varying freezing temperatures and time.

### Morphology of GelMA Hydrogels

**FIGURE 3:**
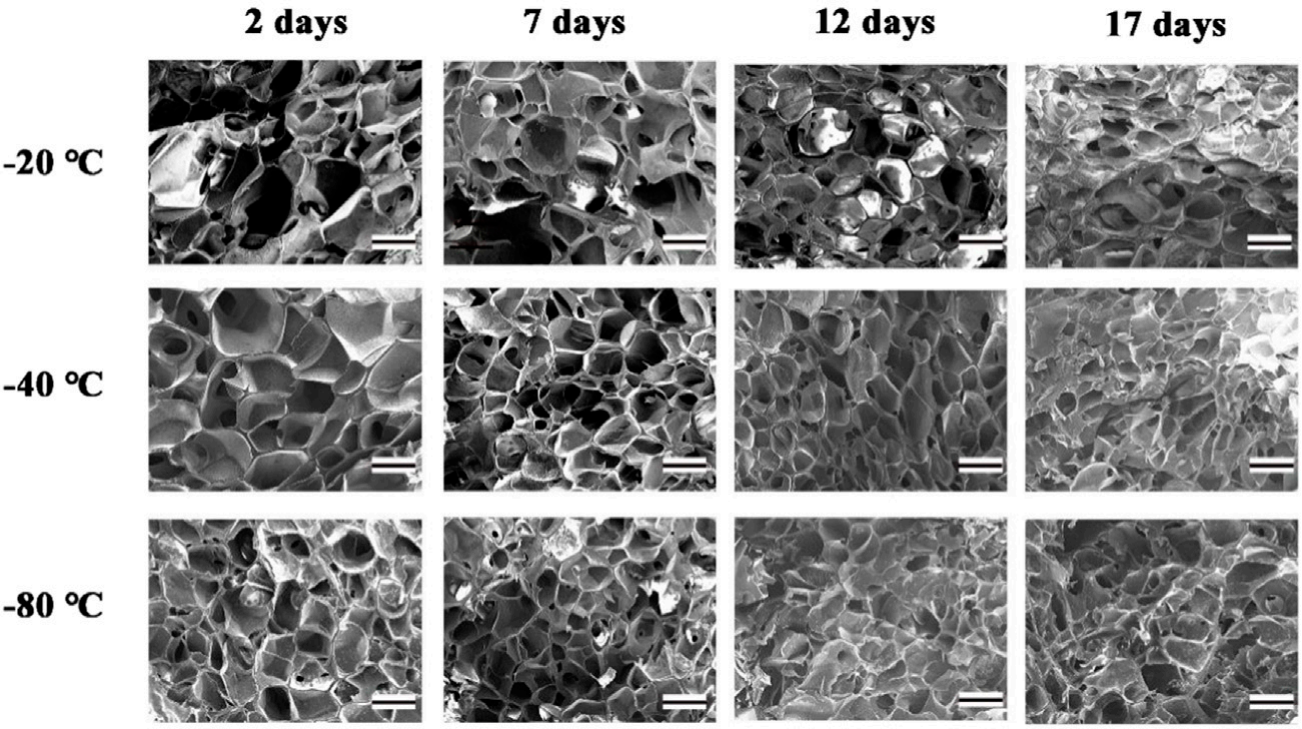
The SEM of GelMA Hydrogels with different freezing temperatures and time. (Scale bars represent 200 µm in all images).

**FIGURE 4:**
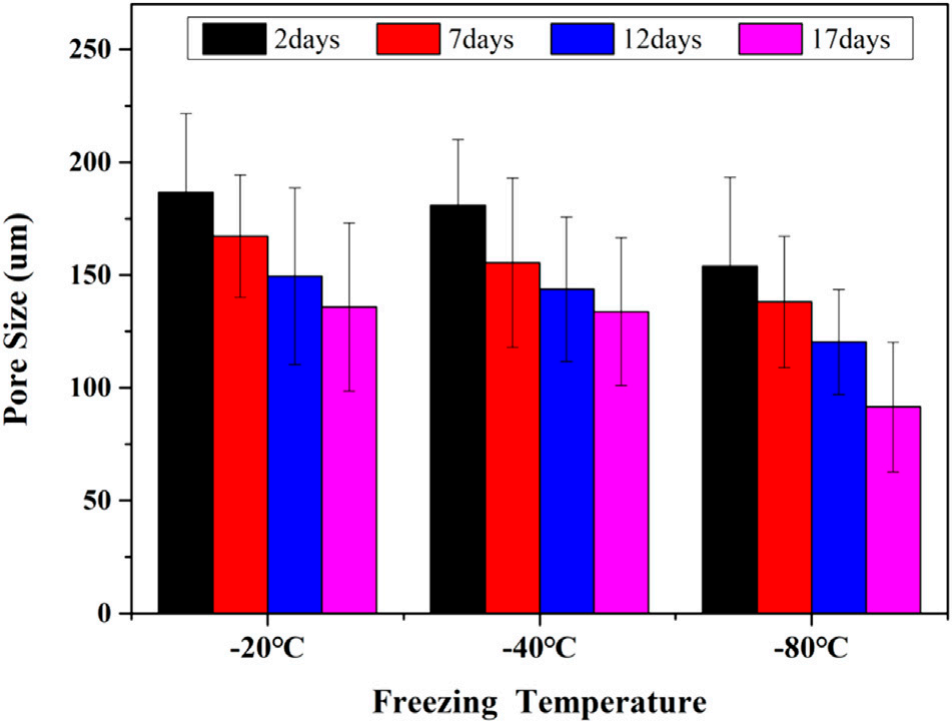
The pore sizes of GelMA Hydrogels with different freezing temperatures and time.

The interconnected pores of scaffolds could enhance cell proliferation by allowing nutrient and oxygen diffusion (Diao et al., 2019). Different pore sizes of porous scaffolds were controlled by different freezing conditions. The surface morphologies of lyophilized GelMA hydrogels with different freezing temperatures (−20°C, −40°C and −80°C) and time (2, 7, 12 and 17 days) are shown in Figure 3. Upon characterization with SEM, the average pore size for each sample was calculated and shown in Figure 4. In fact, some studies have proved that temperature was a key factor to affect the surface morphologies and pore sizes of hydrogels (Kang et al., 1999; Wu et al., 2013).

**FIGURE 5:**
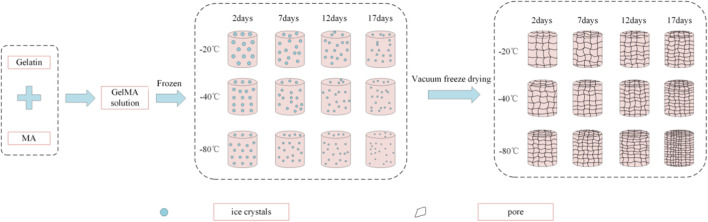
Schematic illustration of the crystallization of GelMA hydrogels under different freezing time and temperatures.

The effect of the freezing temperatures on the pore sizes of GelMA hydrogels was evident that lower temperatures promote smaller pore sizes (Figures 3, 4). When GelMA hydrogels frozen at different temperatures for the same time, GelMA hydrogels frozen at −80°C exhibited smaller pores than hydrogels prepared at −20°C. The morphology and pore sizes of these GelMA hydrogels can be affected by the conditions of freezing temperatures. As showed in Figure 5, during the freezing process, the solvent phase (deionized water) and the polymer phase (GelMA) were separately enriched to form a polymer network (Nam and Park, 1999). In order to reduce the free energy, the polymer phase underwent phase-to-phase migration to a high concentration region over time until a phase equilibrium was reached. The freezing conditions like freezing temperature have great influence on ice crystal morphology. At higher freezing temperature of GelMA hydrogels, slower freezing rate enhanced the formation of larger ice crystals. And the lower undercooling (difference between the freezing temperature and the actual temperature of the material) and the nucleation rate also resulted the larger pore sizes (Ni et al., 2016). At lower freezing temperature, the freezing rate augmented, and the larger undercooling led to the nucleation rate of ice crystals increased. The nucleation rate was larger than the growth rate of ice crystals, leading to the smaller ice crystals (Thiebaud et al., 2002; Ni et al., 2016). Finally, the pores were formed due to the vacuum sublimation during the process of lyophilization.

So, the pore sizes of the scaffolds reduced as the pre-freezing temperature decreased, which was due to that the ice crystals in the scaffolds became smaller as the temperature decreased.

The pore sizes of GelMA hydrogels are also directly impacted by freezing time. At the same temperature, the lower the freezing time, the higher the pore sizes of GelMA hydrogels. Upon characterization with SEM, the average pore size for each sample was calculated and shown in Figures 3, 4. The average pore sizes of the GelMA hydrogels were 186.64 ± 34.96, 167.18 ± 27.16, 149.47 ± 39.12, and 135.80 ± 37.26 μm freezing at −20°C for 2, 7, 12 and 17 days. In this case, longer freezing time produce smaller pores sizes. Similarly, the pore sizes became smaller with longer freezing time when freezing at −40°C and −80°C. The results showed that the pore sizes of GelMA hydrogels could been controlled by freezing time, except for freezing temperatures.

### Swelling Ratio and Mechanical Properties

**FIGURE 6:**
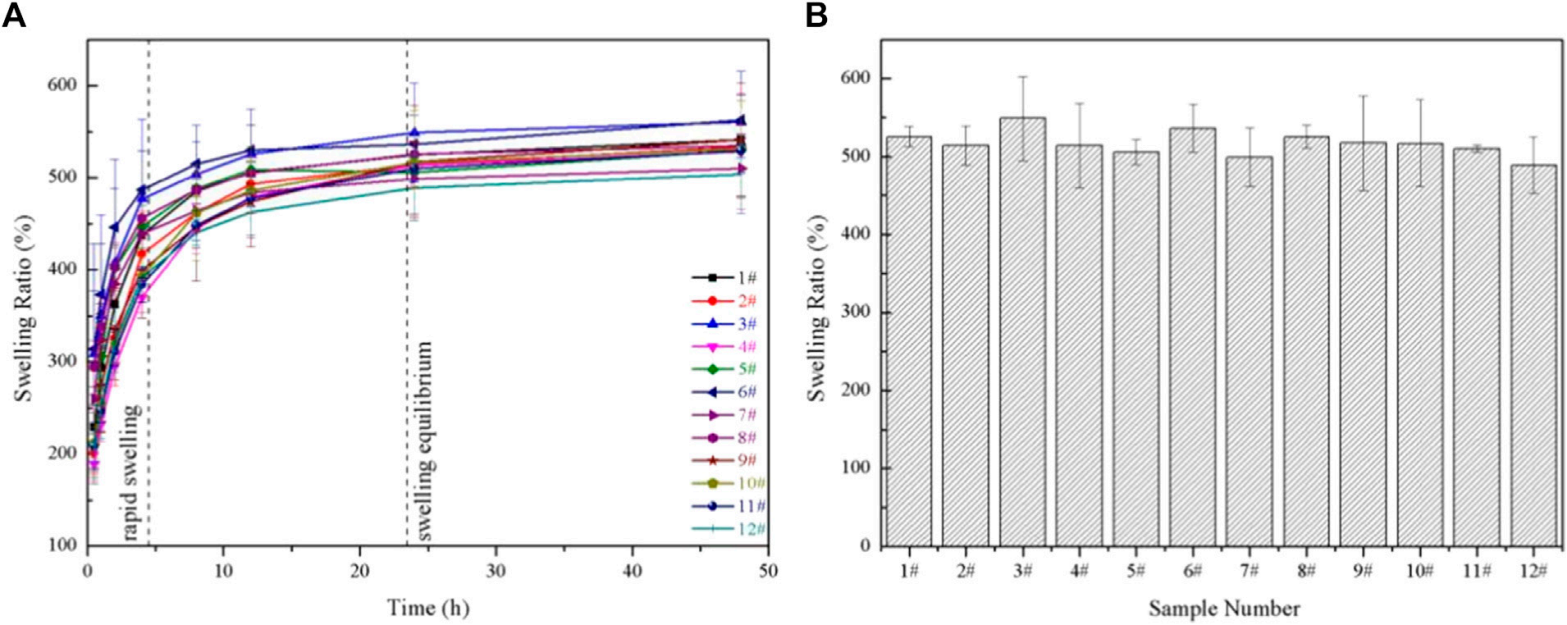
**(A)** The swelling ratio of GelMA hydrogels with different freezing temperatures and time; **(B)** The equilibrium swelling ratio of GelMA hydrogels with different freezing temperatures and time in swelling 24 h.

The swelling properties of GelMA hydrogels depend on the pore sizes, the methacrylation degree, the amount of photoinitiator, and the solvent types (Rahali et al., 2017). It had been demonstrated in this work that the pore sizes of GelMA hydrogels treated in different conditions were different, so, the swelling properties were further studied. The swelling properties of GelMA hydrogels with different freezing temperatures and time are shown in Figure 6. According to Figure 6A, a rapid swelling was observed from 0 to 4 h, and the swelling reached equilibrium swelling after 24 h. The equilibrium swelling ratio in Figure 6B of group 1# - 12# at 24 h were 525.00 ± 13.03%, 514.02 ± 25.14%, 549.04 ± 54.06%, 513.71 ± 54.12%, 505.63 ± 15.75%, 536.27 ± 30.79%, 498.67 ± 37.88%, 525.35 ± 14.69%, 405.08 ± 50.17%, 516.59 ± 55.75%, 510.04 ± 4.59% and 489.06 ± 35.73%, respectively. And there was no significant difference in the equilibrium swelling ratio (*p* > 0.05). The differences in pore sizes in our samples made little contribution to the swelling properties. Therefore, the swelling properties of the GelMA hydrogels were not affected by changing freezing temperatures and time, which may be related to the degree of crosslinking was the same and the number of hydrophilic groups on the scaffolds unchanged.

**FIGURE 7:**
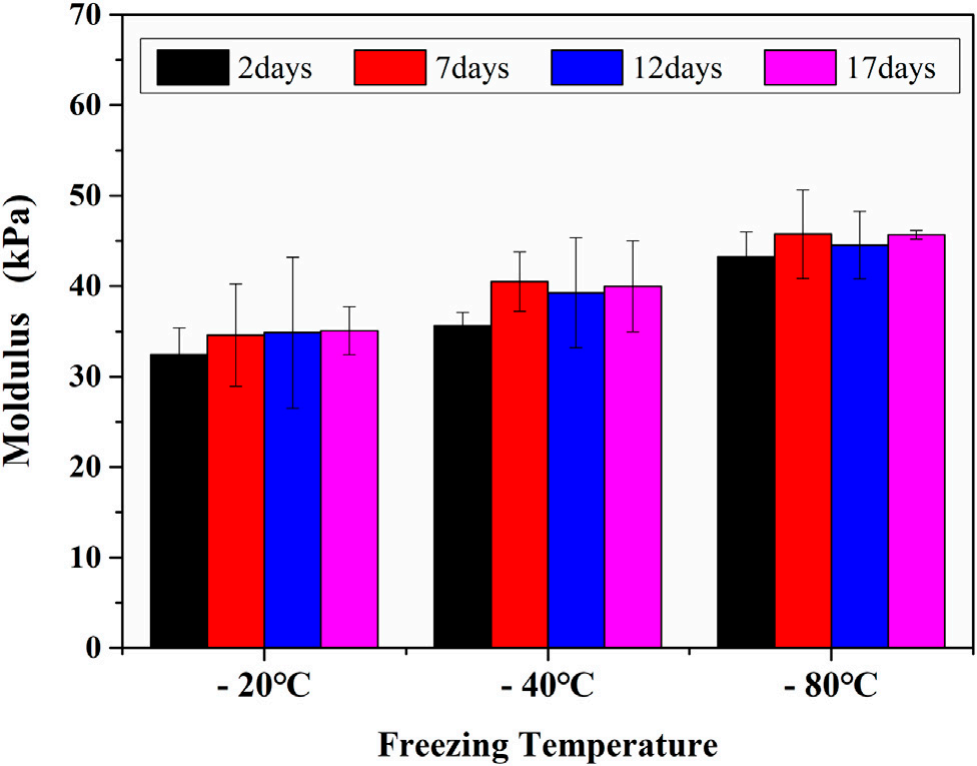
The moldulus of GelMA hydrogels with different freezing temperatures and time.

The compressive properties of the GelMA hydrogel scaffolds treated under different freezing temperatures and time can be seen in Figure 7. The modulus of GelMA hydrogels increased with the reduction of freezing temperatures and the extension of freezing time, which was closely related to the decreased pore sizes of GelMA hydrogel scaffolds. It has been also reported that the mechanical strength of a porous scaffold depends mainly on its pore sizes, and smaller pores were helpful to enhance the biomechanical strength of engineered constructs (Felfel et al., 2019). During the freeze-drying course, the pore sizes decreased with the lower freezing temperatures and longer freezing time, also resulting in the higher compressive strength.

### Growth of MC3T3-E1 Cells in GelMA Hydrogels

**FIGURE 8:**
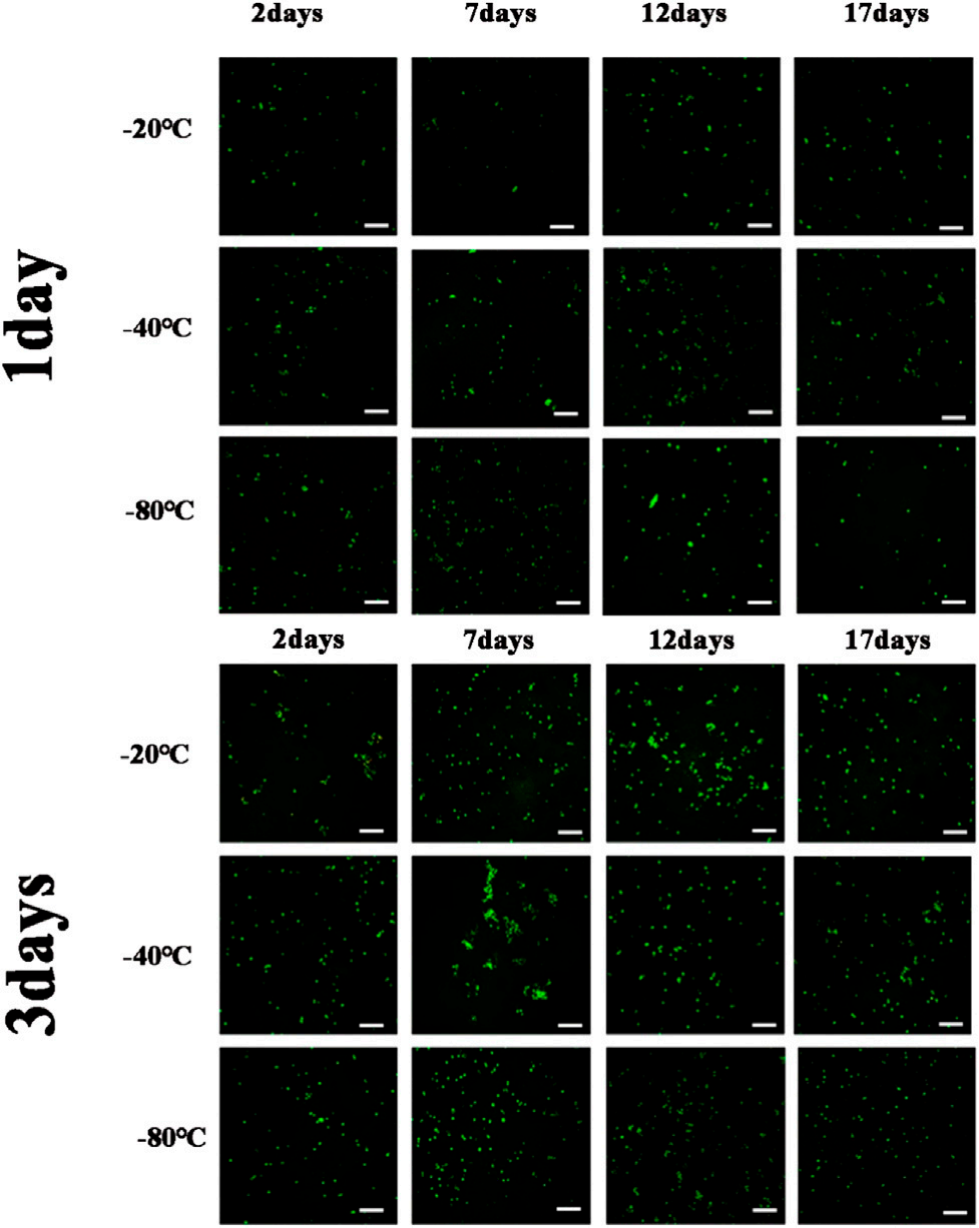
LSCM images of live osteoblasts that were cultured with.

Figure 8 showed the cell viability and proliferation after 1 day and 3 days of cultivation, respectively. Cell viability was determined by green staining for living cells and red for dead cells during culture periods of 1 day and 3 days, and few dead cells were found. The results suggested that GelMA hydrogels could provide a good survival microenvironment for cells due to their ECM-mimetic properties (Monteiro et al., 2020). Consequently, the cell viability of long-term cultures with GelMA hydrogels was assumed to be perfect. As for GelMA hydrogels with larger pore sizes, the cell proliferation rate observed was lower than that observed with the smaller pore sizes. Because of MC3T3-E1 cells could only respond to submicron-scaled pore sizes (Zhang et al., 2017), MC3T3-E1 cells had better cell activity and cell proliferation as the pore sizes of GelMA hydrogels decreased for different freezing time and temperatures. As a result, by controlling the freezing conditions of GelMA hydrogels, the microenvironment suitable for MC3T3 cell was structured. Considering the different cells were suitable for different pore sizes, the tunable pore size hydrogels undoubtedly showed the broad prospect for 3D microenvironment of different types of cells.

GelMA hydrogels with different freezing temperatures and times at 1 day, 3 days (Scale bars represent 100 µm in all images).

## Conclusion

GelMA hydrogels were synthesized with different freezing temperatures and time in this study, and the component, morphology, swelling ratio, mechanical properties and cell adhesion properties of the hydrogels were studied in detail. The pore sizes of the scaffold reduced as the pre-freezing temperature decreased and the freezing time increased. In addition, smaller pore sizes leading to higher MC3T3-E1cell proliferation rate. These results provided a broad prospect for synthesis of GelMA hydrogels with desired pore sizes as well as the appropriate pore sizes for different cells culture.

## Data Availability

The original contributions presented in the study are included in the article/Supplementary Material, further inquiries can be directed to the corresponding authors.
